# Recurrence patterns following nephrectomy for renal cell carcinoma in a Danish nationwide cohort

**DOI:** 10.1002/bco2.375

**Published:** 2024-06-10

**Authors:** Goran Bencina, Rolf Billeskov, Rasmine Bak, Ahmed Al‐Sabbagh, Julie Højgaard Pedersen, Marina Lunetcas, Emma Heeno, Sara Tolouee, Tuba Ashraf, Niels Fristrup, Nessn Azawi

**Affiliations:** ^1^ MSD Madrid Spain; ^2^ MSD Copenhagen Denmark; ^3^ Department of Urology Aarhus University Hospital Aarhus Denmark; ^4^ Department of Urology Aalborg University Hospital Aalborg Denmark; ^5^ Department of Urology Herlev and Gentofte University Hospital Herlev Denmark; ^6^ Department of Urology Odense University Hospital Odense Denmark; ^7^ Department of Urology Rigshospitalet Copenhagen Denmark; ^8^ Department of Urology Zealand University Hospital Roskilde Denmark; ^9^ Department of Oncology Aarhus University Hospital Aarhus Denmark

**Keywords:** carcinoma, renal cell, KEYNOTE‐564 trial, neoplasm metastasis, neoplasm recurrence, local, nephrectomy

## Abstract

**Objectives:**

This study aimed to characterize the demographic and clinical features of patients with renal cell carcinoma (RCC) post‐surgery for localized or locally advanced disease in a national Danish cohort, with a specific focus on describing recurrence patterns in a subgroup aligned with the adjuvant KEYNOTE‐564 trial classification.

**Methods:**

This was a retrospective analysis of the Danish Renal Cancer (DaRenCa) database. Eligible subjects were individuals with an RCC diagnosis between January 2014 and December 2017 who subsequently underwent radical or partial nephrectomy. Variables of interest were demographic and clinical characteristics, rates and sites of recurrence. Recurrence rates were also assessed in a subpopulation stratified using the risk classifications of the KEYNOTE‐564 trial.

**Results:**

A total of 2164 RCC patients were identified. Most patients (84.8%) had non‐metastatic RCC (stage M0). A recurrence was observed in 250 of the M0 patients (13.6%). Patients with a recurrence were older, male, had a higher tumour stage, had undergone radical nephrectomy and had a higher Leibovich score. The majority (74.8%) of M0 patients had recurrence at distant metastatic sites. A total of 392 patients were stratified by the KEYNOTE‐564 risk classification: 335 intermediate‐high risk, 17 high risk and 40 M1 NED (metastatic with no evidence of disease). Recurrence was observed in 37.0%, 88.2% and 27.5% of these risk groups, respectively.

**Conclusions:**

This study elucidates the rates and determinants of post‐surgical RCC recurrence in Denmark, underscoring the potential of adjuvant immunotherapy in refining therapeutic strategies, identifying suitable beneficiaries and minimizing overtreatment risks in RCC care.

## 1 INTRODUCTION

Kidney cancer represents a significant health challenge globally, with the World Health Organization reporting over 430 000 new cases and approximately 175 000 deaths in 2020.^1^, ^2^ Northern Europe, including Denmark, exhibits one of the highest incidence rates of this disease worldwide.^3^, ^4^ Renal cell carcinoma (RCC), the most common form of kidney cancer,^5^ often presents as a localized disease^6^ amenable to surgical intervention.^7^ Despite high survival rates following surgery, the recurrence of RCC is not uncommon and poses a considerable clinical and socio‐economic impact, as well as a less favourable outlook for patients.^8^

The risk of RCC recurrence can be estimated using various prognostic factors,^9^, ^10^ yet the medical community has not reached a consensus on a single, most accurate predictive algorithm among the numerous available. In Denmark, the Leibovich scoring system is commonly used to stratify patients post‐nephrectomy, which has demonstrated varying recurrence rates based on the score categories.^9^, ^11^, ^12^, ^13^ The management options for locally recurrent RCC remain limited, prompting the search for adjuvant therapies to prevent or delay recurrence after nephrectomy.^5^, ^14^, ^15^, ^16^ Pembrolizumab, a programmed death 1 (PD‐1) inhibitor, has recently been approved in the United States and Europe for adjuvant treatment in RCC patients at increased risk of recurrence, based on the results of the KEYNOTE‐564 trial showing significant delay in recurrence in patients treated with adjuvant pembrolizumab (hazard ratio for disease‐free survival 0.68 [95% confidence interval, CI, 0.53–0.87] and 0.63 [95% CI 0.50–0.80] with 24 and 30 months of follow‐up, respectively).^17^, ^18^

Considering the evolving treatment landscape for localized RCC, it is crucial to understand real‐world recurrence patterns across different risk groups. This knowledge can help identify patients who may benefit most from emerging adjuvant therapies. Therefore, the objective of this study was to comprehensively describe the demographic and clinical characteristics of RCC patients following surgery for localized or locally advanced disease in a national Danish cohort, with particular attention to analysing recurrence patterns. Additionally, the study aimed to characterize a subgroup of patients fitting the risk categories utilized for patient inclusion in the adjuvant KEYNOTE‐564 trial classification.

## 2 MATERIALS AND METHODS

### 2.1 Study design and data source

We conducted a retrospective analysis using the Danish Renal Cancer (DaRenCa) database, a comprehensive national clinical quality database. The DaRenCa database integrates data from multiple Danish health registries and is supplemented by manual reviews of medical records. It includes demographic, clinical and treatment information for patients with RCC diagnosed from 2014 to 2017. Follow‐up data collection extended through August 2022, with a median follow‐up of 4.95 years for non‐metastatic patients at diagnosis. The study was approved by the Danish Patient Safety Authority (3‐3013‐2902/1) and complied with the Danish Data Protection Agency regulations (REG‐041‐2021).

### 2.2 Study population

Eligible individuals were those diagnosed with RCC and had undergone radical or partial nephrectomy between 1 January 2014 and 31 December 2017. Exclusion criteria included metastatic disease at diagnosis (except where specified in order to include the ‘M1 no evidence of disease [NED]’ risk category from KEYNOTE‐564), other primary cancers before nephrectomy and insufficient follow‐up or incomplete data.

### 2.3 Study variables and outcomes

The primary outcome was the recurrence of RCC, categorized as either locoregional or distant metastasis. In alignment with the guidelines established by the Danish Renal Cancer Group, recurrence was defined as radiological or pathological evidence of RCC that manifests more than 120 days post‐surgery. This specific time frame is utilized to differentiate between residual disease not completely addressed by surgery and the true recurrence of the disease. Patients presenting with signs of metastatic disease within the first 120 days post‐surgery were considered to have primary metastatic disease and were excluded from the recurrence analysis.

### 2.4 Statistical analysis

We described the demographic and clinical characteristics of all RCC patients (*N* = 2164) and performed a detailed analysis of the group with non‐metastatic disease at baseline (*n* = 1835). Recurrence rates were calculated using Kaplan–Meier analysis at 3 and 5 years post‐surgery. A separate analysis was conducted focusing on the patients meeting the KEYNOTE‐564 trial risk criteria (*n* = 392), assessing recurrence rates and characteristics within defined risk subgroups.

## 3 RESULTS

### 3.1 Description of the RCC cohort

Upon applying the inclusion criteria, we identified 2164 patients with RCC (Table 1). The median age was 66.6 years (interquartile range [IQR] 57.9–72.9), with 65.6% being male and 65.0% having an Eastern Cooperative Oncology Group (ECOG) performance status of 0. A substantial proportion of patients (*n* = 1835; 84.8%) had non‐metastatic (M0) disease at diagnosis. Within this M0 cohort, 69.4% had an ECOG performance status of 0, 71.7% were diagnosed at tumour stage T1, 78.8% had node stage Nx and 74.4% had a clear cell RCC subtype (Table 2). The median Fuhrman grade among the M0 cohort was 2 (IQR 2–3), and the majority of patients (58.7%) had a Leibovich score of 0–2, indicating low risk of recurrence.

**TABLE 1:** Demographic and clinical characteristics of patients with renal cell carcinoma, 2014–2017.

	Study population (*N* = 2164)
Age at diagnosis	
Mean years (SD)	65.1 (10.9)
Median years (IQR)	66.6 (57.9–72.9)
Males	1460 (65.6%)
Body mass index, mean (SD)	28.11 (5.92)
M stage	
M1	322 (14.9%)
M0	1835 (84.8%)
Unknown	7 (0.3%)
Smoking history	
Current smoker	553 (25.6%)
Former smoker	702 (32.4%)
Never smoker	894 (41.3%)
Unknown	15 (0.7%)
ECOG performance status	
0	1406 (65.0%)
1	539 (24.9%)
2+	211 (9.7%)
Unknown	8 (0.4%)

*Note*: Values are presented as *n* (%) unless otherwise specified.

Abbreviations: ECOG, Eastern Cooperative Oncology Group; IQR, interquartile range; SD, standard deviation.

**TABLE 2:** Clinical characteristics of patients with primary non‐metastatic renal cell carcinoma.

	Subgroup with M0 (*N* = 1835)
ECOG performance status	
0	1274 (69.4%)
1	422 (23.0%)
2+	137 (7.4%)
Unknown	2 (0.1%)
Body mass index, mean (SD)	28.35 (6.00)
T stage at diagnosis	
T1	1315 (71.7%)
T2	131 (7.1%)
T3	319 (17.4%)
T4	7 (0.4%)
Missing	63 (3.4%)
N stage	
Lymph node positive (N1)	10 (0.5%)
Lymph node negative (N0)	108 (5.9%)
Lymphadenectomy not performed (Nx)	1446 (78.8%)
Missing	271 (14.8%)
Histology	
Clear cell	1366 (74.4%)
Papillary	294 (16.0%)
Chromophobe	120 (6.5%)
Other	47 (2.6%)
Missing	8 (0.4%)
Sarcomatoid features	54 (2.9%)
Fuhrman grade	
1	290 (15.8%)
2	924 (50.4%)
3	334 (18.2%)
4	122 (6.6%)
Unknown	165 (9.0%)
Leibovich score	
Low risk (0–2)	1077 (58.7%)
Intermediate risk (3–5)	269 (14.7%)
High risk (>5)	309 (16.8%)
Unknown	180 (9.8%)

*Note*: Values are presented as *n* (%) unless otherwise specified.

Abbreviations: ECOG, Eastern Cooperative Oncology Group; SD, standard deviation.

### 3.2 Rates and sites of recurrence

**FIGURE 1:**
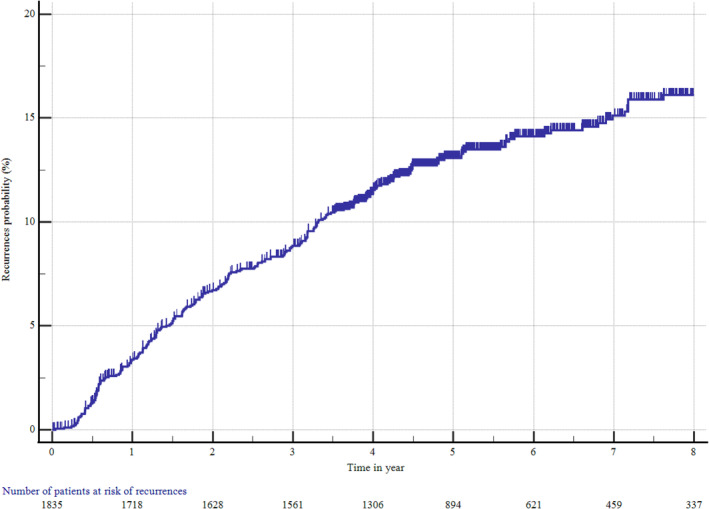
Kaplan–Meier plot of recurrence in the M0 cohort (*n* = 1835).

In the M0 cohort, RCC recurrence was observed in 250 patients (13.6%) during the study period. Kaplan–Meier analysis revealed recurrence rates of 8.9% (95% CI 7.5–10.4) at 3 years and 13.1% (95% CI 11.5–14.4) at 5 years post‐nephrectomy (Figure 1 and Table S1). Recurrence rates varied by tumour stage and Leibovich score (Figure S1). Patients with a recurrence had older age (median [IQR] of 66.8 [58.6–74.4] vs. 65.8 [57.3–72.5] years for patients with and without recurrences, respectively), male sex (72.0% vs. 62.7%), radical nephrectomy (54.0% vs. 43.4%) and higher tumour stage and Leibovich score (Figure S1) compared with patients without recurrence. Locoregional recurrence occurred in 25.2% of patients, while the remaining 74.8% experienced distant metastases, with or without concurrent local recurrence (Table S2). The lungs (43.6%) and bones (18.8%) were the most common sites of distant recurrence in M0 patients.

### 3.3 Analysis of patients stratified by KEYNOTE‐564 risk classification

**FIGURE 2:**
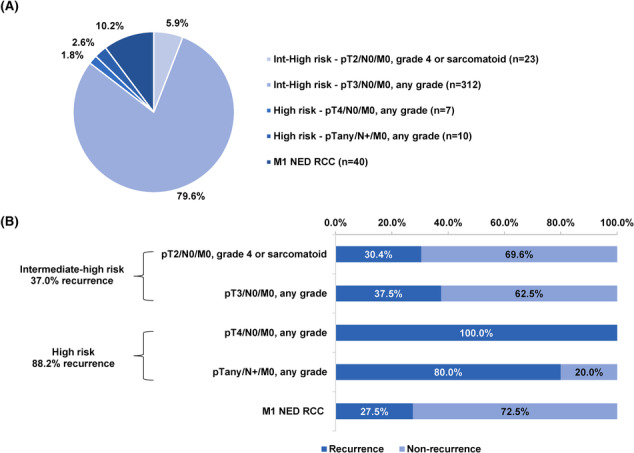
Recurrence in patients stratified by the KEYNOTE‐564 classification. (A) Distribution of 392 patients among the various KEYNOTE‐564 risk categories. The categories are described in detail in Section 2. (B) Rates of recurrence within each risk category. Int, intermediate; NED, no evidence of disease; RCC, renal cell carcinoma.

**FIGURE 3:**
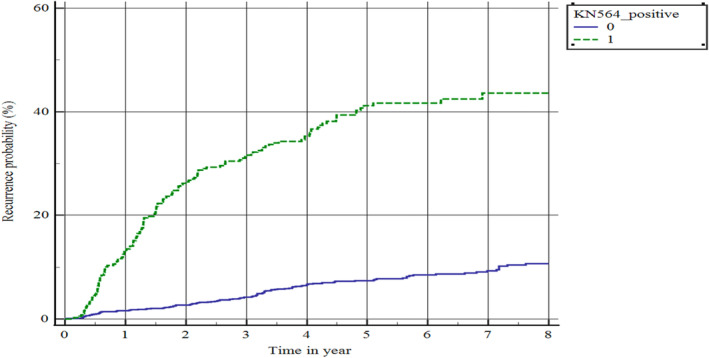
Kaplan–Meier plot of recurrence in the patients stratified by KEYNOTE‐564 risk classification (*n* = 392). The green line represents the 392 patients who fit the KEYNOTE‐564 risk criteria. The blue line represents the remaining 1443 patients who did not meet the KEYNOTE‐564 risk criteria.

**FIGURE 4:**
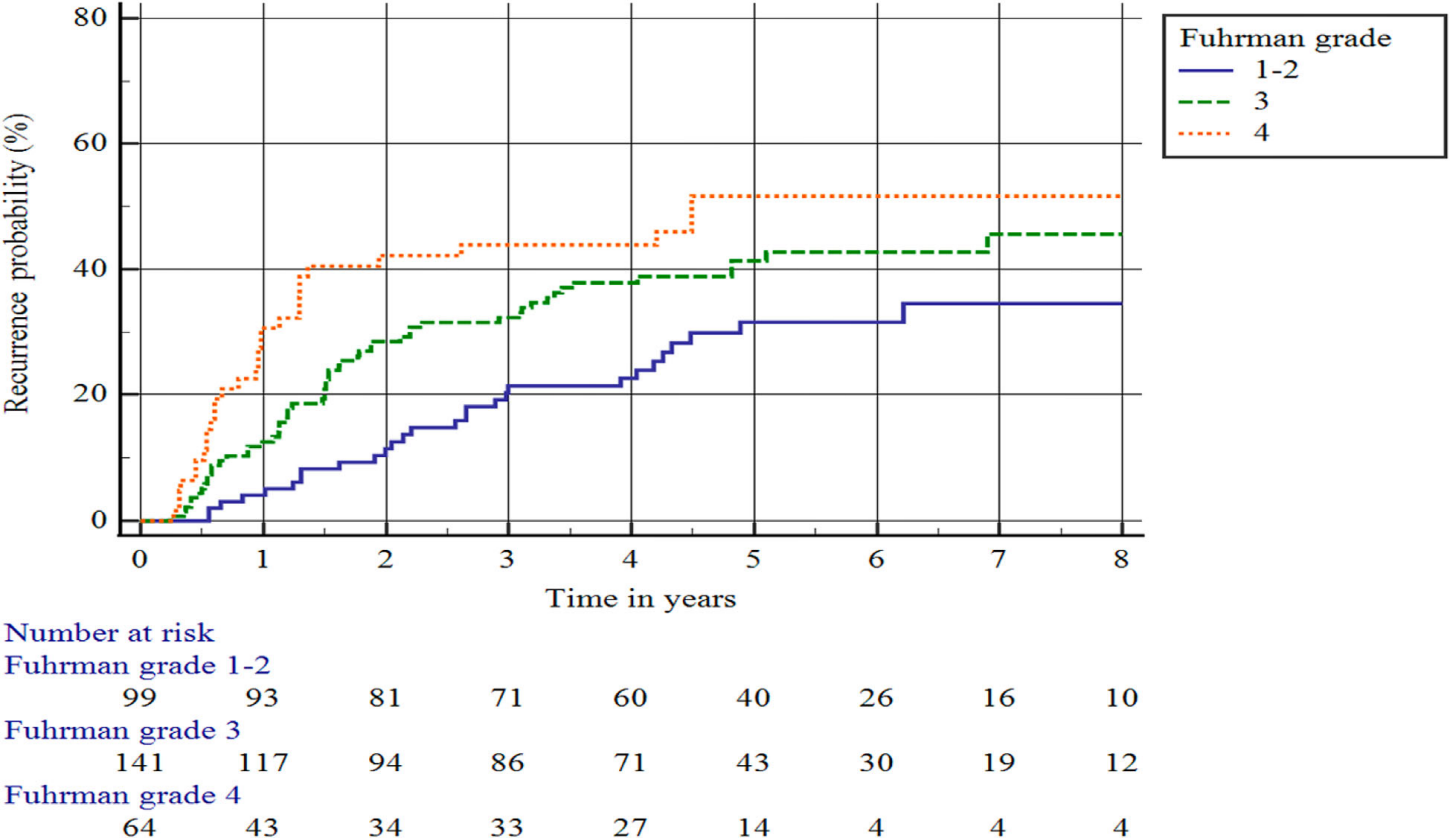
Kaplan–Meier plot of recurrence in the KEYNOTE‐564 subpopulation of pT3/N0/M0 patients stratified by Fuhrman grade.

Of the patients stratified by KEYNOTE‐564 risk criteria (*n* = 392), 335 (85.5%) were at intermediate‐high risk (T2 grade 4/sarcomatoid or T3N0M0), 17 (4.3%) were at high risk (T4N0M0 or N1) and 40 (10.2%) were classified as M1 NED (Figure 2A). Kaplan–Meier analysis showed 3‐ and 5‐year recurrence rates of the entire KEYNOTE‐564 population of 30.4% and 40.1%, respectively (Figure 3 and Table S1). Further stratification of the large and heterogenous pT3/N0/M0 patients revealed 3‐/5‐year recurrence rates of 20.4%/31% for Fuhrman grades 1–2, 31.9%/42% for grade 3 and 43.7%/52% for grade 4 (Figure 4 and Table S1). For the 11 M1 NED patients who experienced recurrence, the most common sites were the lungs (50.0%), bones (41.7%) and kidney (16.7%; Table S2).

## 4 DISCUSSION

Our analysis provides a comprehensive characterization of Danish patients undergoing surgery for RCC and describes the distribution of variables in patients with and without disease recurrence. By employing the risk criteria utilized to include patients in the adjuvant pembrolizumab trial (KEYNOTE‐564), we stratified a subgroup of patients and demonstrated an increased rate of recurrence corresponding to higher prognostic risk levels.

The observed 3‐ and 5‐year recurrence rates of 8.9% (95% CI 7.5–10.4) and 13.1% (95% CI 11.5–14.4) post‐nephrectomy among M0 patients across all T stages align with previously reported rates.^9^, ^12^, ^19^, ^20^, ^21^, ^22^, ^23^, ^24^ Recurrence rates are influenced by various factors, including disease status at baseline, surgical approach and the specific definitions of outcomes used in different studies. Our findings are comparable to those reported by Azawi et al.,^13^ Leibovich et al.^9^ and Marconi et al.,^24^ who investigated similar patient populations.

A significant proportion of recurrences in our cohort occurred at distant sites (74.8%), with the remainder being local. This distribution is consistent with some previous studies,^19^, ^25^ although others have reported a higher incidence of distant recurrences, closer to 90%.^8^, ^22^ We found that recurrence was more frequent in patients with a higher tumour stage and Leibovich score, male patients and patients who underwent radical nephrectomy, which was in line with findings from earlier research.^9^, ^12^, ^26^

A key aspect of our study was the application of risk stratification criteria from the KEYNOTE‐564 trial, allowing us to assess whether our data were consistent with the trial's findings. The majority of our patients fell into the intermediate‐high‐risk category (85.5%), similar to the trial's composition (86.9% in the placebo group).^17^ Our descriptive analyses revealed a trend of increasing recurrence rates with higher risk levels among primary M0 patients, mirroring both the pattern observed and absolute recurrence rates in the placebo group of KEYNOTE‐564 for the intermediate‐high‐ and high‐risk patients.^17^

Patients with pT3 disease exhibited a wide range of recurrence rates, with higher recurrence observed with increasing Fuhrman grade. This has been observed previously, although our study reported lower overall 3‐year recurrence rates for pT3/N0/M0 patients compared with those reported from the US SEER database by Sundaram et al.^8^ This may reflect differences in patient demographics and the centralized nature of nephrectomy procedures in Denmark compared with the United States.

Our findings indicate that the tendency for RCC recurrence post‐surgery is a significant concern, with a notable proportion of patients experiencing disease return despite the surgical approach taken. While radical and partial nephrectomies are associated with different recurrence rates, the focus of our study is not on the surgical technique as a risk factor but on the broader patterns of recurrence that emerge following surgery. This distinction is crucial for guiding post‐operative management and surveillance strategies. Understanding the trends in recurrence can help clinicians identify patients who may benefit from more intensive monitoring or adjuvant therapies, potentially improving long‐term outcomes.

The M1 NED group in our study had a lower 3‐year recurrence rate compared with KEYNOTE‐564 (23% vs. > ~65%).^17^ This discrepancy could be attributed to the high mortality risk in M1 NED patients, leading to censorship in our analysis and potentially underestimating the true recurrence risk. Given the small sample size of M1 NED patients in our study (*n* = 40), these results should be interpreted cautiously.

Our study's strengths include its multicentre design and comprehensive inclusion of Danish RCC patients, with complete follow‐up data from registries and detailed information from medical records. However, we acknowledge limitations such as the retrospective nature of the study, the lack of adjustment for partial/radical nephrectomy and confounders like socio‐economic status. Additionally, the absence of certain histological data, such as programmed cell death ligand 1 (PD‐L1) expression, limits further risk stratification.

In conclusion, our real‐world data analysis highlights the potential of adjuvant immunotherapy in managing primary non‐metastatic RCC post‐surgery. By identifying factors that are consistent with those delineated in clinical trials such as KEYNOTE‐564, we can better identify patients who are likely to benefit from this treatment approach, thereby enhancing therapeutic precision and reducing the risk of overtreatment. These insights affirm the role of adjuvant immunotherapy as a valuable strategy for managing RCC recurrence risks.

## AUTHOR CONTRIBUTIONS

**Goran Bencina:** Writing—original draft; writing—review and editing. **Rolf Billeskov:** Writing—original draft; writing—review and editing. **Rasmine Bak:** Data curation; writing—review and editing. Ahmed **Al‐Sabbagh:** Data curation; writing—review and editing. **Julie Højgaard Pedersen:** Data curation; writing—review and editing. **Marina Lunetcas:** Data curation; writing—review and editing. **Emma Heeno:** Data curation; writing—review and editing. **Sara Tolouee:** Data curation; writing—review and editing. **Tuba Ashraf:** Data curation; writing—review and editing. **Niels Fristrup:** Writing—original draft; writing—review and editing; supervision. **Nessn Azawi:** Conceptualization; data curation; formal analysis; methodology; writing—original draft; writing—review and editing; supervision.

## CONFLICT OF INTEREST STATEMENT

Goran Bencina and Rolf Billeskov are employees of Merck Sharp & Dohme LLC, a subsidiary of Merck & Co., Inc., Rahway, NJ, USA, and may own stocks and/or stock options in Merck & Co., Inc. Niels Fristrup has performed educational sessions for Bristol‐Myers Squibb Company, AstraZeneca, Pfizer and Merck and Co., Inc. and has performed consultancy for Merck and Co., Inc., Eisai and Ipsen. All other authors have no conflicts of interest to report.

## Supporting information


**Table S1.** 3‐ and 5‐year recurrence rates in the M0 cohort as well as KEYNOTE‐564 subpopulations by Kaplan–Meier analysis^a^.
**Table S2.** Site of recurrence of renal cell carcinoma among patients with recurrence, by initial diagnosis^a^.
**Figure S1.** Recurrence rates in patients with non‐metastatic renal cell carcinoma.
