# Supplementary material for: Recurrence patterns following nephrectomy for renal cell carcinoma in a Danish nationwide cohort
Source: BJUI Compass. 2024 Jun 10;5(8):791–8. doi: 10.1002/bco2.375 (PMC11327494; doi:10.1002/bco2.375)
Supplement: Supplementary file 1 — Table S1. 3‐ and 5‐year recurrence rates in the M0 cohort as well as KEYNOTE‐564 subpopulations by Kaplan–Meier analysisa. Table S2. Site of recurrence of renal cell carcinoma among patients with recurrence, by initial diagnosisa. Figure S1. Recurrence rates in patients with non‐metastatic renal cell carcinoma. [file BCO2-5-791-s001.docx]

**Supplementary Table 1. 3- and 5-year recurrence rates in the M0 cohort as well as KEYNOTE-564 subpopulations by Kaplan-Meier analysis^a^**

| Population | No. patients | 3-year recurrence rate | 5-year recurrence rate |
| --- | --- | --- | --- |
| M0 | 1,835 | 8.9% (95% CI 7.5-10.4) | 13.1% (95% CI 14.4-11.5) |
| KN564 (all) | 392 | 30.4% | 40.1% |
| KN564 Intermediate-high risk | 335 |  |  |
| pT2/N0/M0 gr4/ sarcomatoid | 23 | 22.75% (95% CI 5-40) | 33% (95% CI 13-53) |
| pT3/N0/M0 any grade | 312 | 30.5% (95% CI 26-36) | 41% (95% CI 34-46) |
| Fuhrman grade 1-2 | 99 | 20.4% (95% CI 13-28) | 31% (95% CI 21.5-37.5) |
| Fuhrman grade 3 | 141 | 31.9% (95% CI 24-39) | 42% (95% CI 33-52) |
| Fuhrman grade 4 | 64 | 43.7% (95% CI 30-56) | 52% (95% CI 38-63) |
| KN564 High risk | 17 |  |  |
| pT4/N0/M0 any grade | 7 | 66% (95% CI 40-100) | N/A^b^ |
| pTany/N+/M0 any grade | 10 | 82% (95% CI 55-100) | 82% (95% CI 55-100) |
| M1 NED | 40 | 23% (95% CI 8-37) | 37% (95% CI 16-53) |

Abbreviations: CI, confidence interval; KN, KEYNOTE; N/A, not available

^a^ Please see note for Kaplan-Meier analysis in the Materials and Methods section regarding censoring of deceased patients with no registered recurrence which can result in slightly higher recurrence rates compared to Supplementary Table 2, Figure 1, and Supplementary Figure 1.

^b^ For T4 in particular, all patients were deceased 5 years post-nephrectomy, and hence 5-year recurrence rate was 0%.

Supplementary Table 2. Site of recurrence of renal cell carcinoma among patients with recurrence, by initial diagnosis^a^

|  | Primary M0  (N=250) | Primary M1 NED (N=11) |
| --- | --- | --- |
| Local recurrence (only) | 63 (25.2%) | 2 (16.7%) |
| Lung+ | 109 (43.6%) | 6 (50.0%) |
| Liver+ | 21 (8.4%) | 0 (0.0%) |
| Bone+ | 47 (18.8%) | 5 (41.7%) |
| Brain+ | 13 (5.2%) | 1 (8.3%) |
| Other+ | 124 (49.6%) | 8 (66.7%) |

Abbreviations: M0, primary non-metastatic renal cell carcinoma; M1 NED, metastatic with no evidence of disease

^a^ Values are presented as n (column %). Patients could have more than one site of metastasis, and thus the totals do not sum to 100%.

Supplementary Figure 1. Recurrence rates in patients with non-metastatic renal cell carcinoma

Abbreviations: Int, intermediate

The bars indicate the percentage of M0 patients within each subcategory that exhibited recurrence as defined in the Materials and Methods text.
